# Demographic, morphologic, hormonal and metabolic factors associated with the rate of improvement from equine hyperinsulinaemia-associated laminitis

**DOI:** 10.1186/s12917-022-03149-z

**Published:** 2022-01-18

**Authors:** Martin Sillence, Alexandra Meier, Melody de Laat, Rebecca Klee, Dania Reiche

**Affiliations:** 1 Queensland University of Technology (QUT), School of Biology and Environmental Science, Brisbane, Queensland 4000 Australia; 2 Boehringer Ingelheim Vetmedica GmbH, Ingelheim am Rhein, Germany

**Keywords:** Insulin, Endocrinopathic, Equine metabolic syndrome, Diagnosis, Recovery

## Abstract

**Background:**

Although several studies have investigated factors associated with the onset and occurrence of hyperinsulinaemia-associated laminitis (HAL), few have examined the factors associated with the rate of improvement during recovery from an acute bout of the disease. This observational study sought to discover if a range of demographic, morphologic, hormonal and metabolic variables are associated with the improvement rate from HAL in 37 naturally-occurring cases identified by 16 clinics across Germany. Each case was evaluated for laminitis severity on the day of inclusion in the trial (d 0), then after 4, 9, 14, 25 and 42 d. The horses were managed according to best clinical practice including restricting exercise and prescribing a diet of hay-only, for a minimum of 9 d. Blood samples were also collected during each evaluation, except on d 9, and analysed for glucose, insulin, ACTH and leptin.

**Results:**

Based on individual clinical laminitis scores plotted against time, most horses improved markedly within 2 weeks, with a ‘fast group’ (*n* = 27) having a median (interquartile range) score on a 12-point scale of 0 (0–2) by d 14. However, there was a clear disparity within the total cohort, as ~ 1 in 4 horses demonstrated much slower improvement, with a median score of 5 (4–7) by d 14, or a marked relapse thereafter (‘slow group’, *n* = 10). Horses in the slow improvement group were younger (12.5 (8.8–16.3) vs 17 (14–24) yr; *P* = 0.008), but were not more likely to be heavier, male, very fat, to have presented with a previous history of laminitis or elevated ACTH concentrations, or to be receiving pergolide treatment. Of the hormonal and metabolic parameters measured, glucose and insulin concentrations were within the normal range following transition to the hay-only diet, but were higher in the group that failed to improve quickly, with a small but significant difference being evident on d 4, 14 and 25 for glucose (11 to 16%; *P* < 0.05), and a larger difference for insulin on d 14 and 25 (51 to 55%; *P* < 0.05). There was no difference between the groups in ACTH or leptin concentrations throughout the study. The main limitations of this study were the small number of slow-improvement horses and an inability to control or measure certain variables, such as feed quality.

**Conclusions:**

Young age and a modest increase in blood glucose and insulin concentrations are associated with delayed laminitis improvement.

## Background

Laminitis is a condition of the horse’s foot characterised by a failure of the connective tissue (or lamellae), which normally anchors the pedal bone to the hoof wall [1]. The onset and recurrence of the condition have several triggers, but pasture-associated endocrinopathic laminitis has become the most prevalent form, accounting for 89% of cases in one study [2]. Endocrinopathic laminitis is a serious condition, with a Danish study showing that 33% of cases were euthanized within 12 months of diagnosis [3], and a global survey revealing that the condition recurs in 34% of survivors within 2 years [4].

Over the past 30 years, it has been demonstrated that insulin dysregulation (ID) is a central underlying problem in endocrinopathic laminitis [5–7]. In particular, when horses or ponies that suffer ID graze on pasture or other feeds that have a high content of non-structural carbohydrates, blood glucose concentrations increase and hyperinsulinaemia can ensue, sometimes associated with incretin hormones such as glucagon-like peptide-1 (GLP-1) [8]. Extreme and/or prolonged hyperinsulinaemia can induce laminitis, as shown experimentally more than a decade ago [6, 9]. Meanwhile, as no studies have produced evidence for a causative effect of other hormones originally associated with the condition, such as cortisol or ACTH, endocrinopathic laminitis may be described more accurately as hyperinsulinaemia-associated laminitis (HAL) [10].

The breeds that tend to be predisposed to HAL, e.g. Welsh ponies, are often those that are efficient feeders and glucose absorbers, likely through centuries of adaptation to seasonal shortages of feed [11]. Older animals who develop pituitary *pars intermedia* dysfunction (PPID), as characterised by elevated ACTH concentrations, are also susceptible to ID and HAL [12]. Susceptible animals are often overweight, and although obesity per se may have no direct role in laminitis [13], the deposition of fat at particular body sites such as in the neck is strongly associated with ID [14, 15].

Although several studies have improved our understanding of the risk factors and pathogenesis of HAL over recent years, few have examined the factors associated with laminitis recovery. This could be due in part, to the fact that the traditional Obel system, commonly used for grading laminitis, was designed for relatively severe, sepsis-related cases [16], and not to detect the milder form and more subtle changes that are associated with HAL. In a recent clinical study, we modelled the rate and pattern of improvement in 80 cases of naturally-occurring, suspected HAL [17], deploying a modified version of the Obel method which assigns separate scores to five discreet clinical signs and provides an aggregate score on a scale of 0–12 [18]. In that study, we observed two distinct patterns of improvement - some animals showed a rapid improvement, whereas others were slow to improve, or showed various clinical signs that waxed and waned over the observation period [18].

The aim of the present work was to examine a sub-group of these animals with confirmed HAL, to determine if the rate of improvement in clinical signs was associated with any morphologic characteristics such as body weight or fatness, or with changes in hormones that have been associated with laminitis, PPID, or obesity. Such information would be useful to guide recovery and/or rehabilitation strategies, and to offer an evidence-based prognosis for the patient.

## Results

**Fig. 1:**
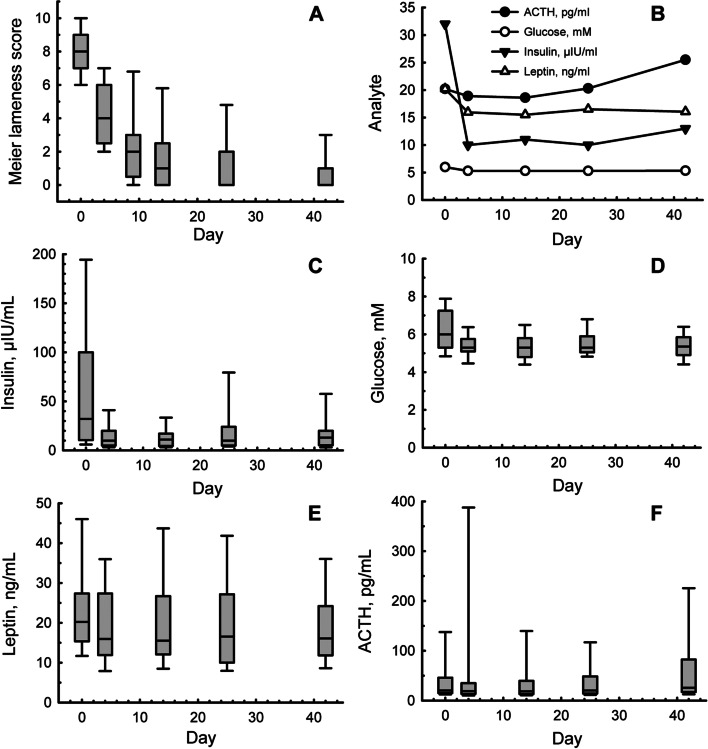
Box and whisker plots showing the median, 10th, 25th, 75th, and 90th percentiles for clinical laminitis scores (**A**) and glucose and hormone concentrations (**B** to **F**) over 42 days during recovery from hyperinsulinaemia-associated laminitis in 37 horses and ponies. Panel B compares the median values for each analyte over time, including insulin, glucose, leptin and ACTH. The large error bars indicate significant disparity within this cohort, which was subsequently partitioned into two groups: those that showed an immediate improvement in clinical signs and those that did not

Thirty-seven cases of ID and HAL were confirmed, from 41 animals that had been selected at random for detailed sampling and analysis, among a total of 80 cases that were enrolled in the original clinical study [18]. The median clinical laminitis scores and analyte concentrations for these 37 animals were plotted against time and are presented in Fig. 1.

**Fig. 2:**
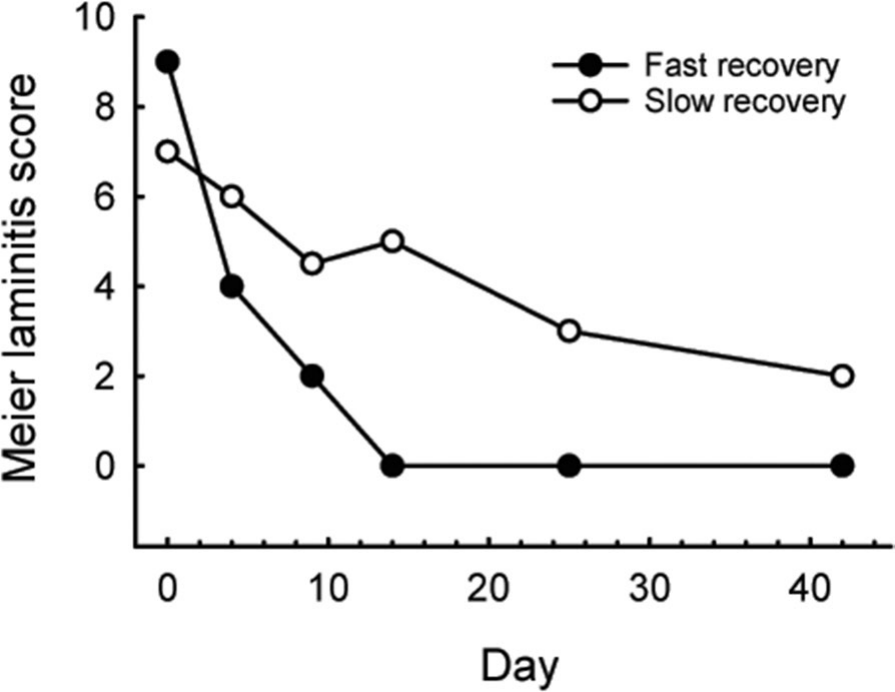
Median clinical laminitis scores measured over 42 days in 27 horses and ponies that recovered quickly and 10 that recovered slowly from hyperinsulinaemia-associated laminitis

The clinical laminitis scores and analyte concentrations showed a large degree of variation at certain time-points, so individual plots were evaluated for each horse. Consistent with our earlier study [18], these plots confirmed that while the pattern of improvement in clinical signs appeared to follow the form of an exponential decrease in most animals (Fig. 1A), 10 horses did not conform to this pattern and did not recover rapidly. The widest separation between the group that improved rapidly and the group that did not, was evident on d 14, when a clinical laminitis score of 4 was identified as a clear “cut off” between the fast and slow groups. At this time, the median (IQR) score of animals in the fast group was 0 (0–2). In contrast, on d 14 the median score in the slow group was 5 (4–7). One horse that appeared to respond well initially, suffered a marked relapse and returned a score of 8 by d 25. This animal was also assigned to the slow improvement group. The pattern of improvement in fast (*n* = 27) and slow (*n* = 10) groups is illustrated in Fig. 2.

A repeated-measures analysis of glucose and hormone concentrations over time for the entire cohort (Fig. 1B), revealed a significant change for ACTH (*P* = 0.04), glucose and insulin (*P* < 0.001), but not for leptin (*P* = 0.146). A *post-hoc* pairwise comparison revealed that both glucose and insulin concentrations were higher on d 0 than on all other days (*P* < 0.05), but were stable thereafter. This might have reflected the change in diet, or the fact that the horses were not fasted before blood collection on d 0. Concentrations of ACTH were only different between d 4 and d 42 (P < 0.05), possibly reflecting a seasonal change in this hormone.

Following the *post-hoc* classification of horses according to their rate of improvement in clinical signs, the data were examined for possible associations with several variables. First, the dietary management of the horses was examined, as shown in Table 1.

**Table 1:** Feed types provided preceding a bout of laminitis and for the following 42 days in horses that showed a fast (*n* = 27) or slow (*n* = 10) improvement in clinical signs. The numerals represent the number of horses fed a particular feed type

Group	Feed type	Day of study				
		−14 to − 1	0 to 9	10 to 14	15 to 25	26 to 42
Fast	Hay	26	27	27	25	26
	Pasture	14	0	5	6	8
	Succulents^a^	13	0	0	0	0
	Concentrates/pellets	6	0	0	2	2
	Forage^b^	7	0	3	3	6
	Grains	5	0	0	0	0
	Silage/haylage	3	0	1	0	1
Slow	Hay	9	10	10	8	10
	Pasture	3	0	0	1	1
	Succulents	3	0	0	0	0
	Concentrates/pellets	3	0	0	0	1
	Forage	2	0	1	2	1
	Grains	4	0	0	0	0
	Silage/haylage	2	0	0	0	0

^a^Mainly carrots, but occasionally other root crops with a high water content

^b^Grass or other plants such as clover or lucerne (alfalfa) cut and dried for fodder

Preceding the bout of laminitis, nearly all the horses had been fed hay, with 28 out of 37 horses having received at least one other feed type. During the recovery period, all the horses received hay only for the first 9 d as prescribed by the study protocol, and while some horses in the fast recovery group received other feed types from d 10 to d 14, 9/10 horses in the slow recovery group remained on hay only during this period. Thus, there is no evidence that the slow recovery of the animals in this group was due to the feeding of high-energy feedstuffs during the recovery period.

The demographic and morphologic characteristics of the horses were compared next, as shown in Table 2.

**Table 2:** Demographic and morphological characteristics of horses according to their rate of recovery from hyperinsulinaemia-associated laminitis

Variable	Fast improvement (***n*** = 27)	Slow improvement (***n*** = 10)	***P*** value
Breeds, n			
Andalusian	1	0	–
Appaloosa	1	0	–
Crossbred	1	0	–
German Riding Pony	4	0	–
Haflinger	2	0	–
Hanoverian	0	3	–
Icelandic	4	2	–
Selle Francais	1	0	–
Shetland Pony	8	2	–
Welsh Pony	2	2	–
Westphalian	0	1	–
Other - Not determined	3	0	–
Age, median (IQR), yr^a^	17 (14–24)	12.5 (8.8–16.3)	0.008^b^
Estimated body weight, median (IQR), kg	391 (232–470)	421 (235–616)	0.374^b^
Males, n (%)	13 (48.1)	2 (20)	0.153^c^
Graded as ‘very fat’ (score 5), n (%)	8 (29.6)	1 (10)	0.393^c^
History of laminitis, n (%)	16 (59.3)	5 (50)	0.716^c^
Receiving pergolide treatment, n (%)	7 (25.9)	2 (20)	1.000^c^
Elevated ACTH, n (%)	11 (40.7)	3 (30)	0.71^c^

^a^Interquartile range

^b^Mann-Whitney Rank Sum Test

^c^Odds-ratio test

Analysis of the frequency of horses and ponies in each group, with a particular characteristic, revealed that the animals in the slow group were more likely to be younger than animals in the fast group (*P* = 0.008). Based on the *P* values for other variables, it was clear that animals in the slow group were not more likely to be male; heavier or very fat; to have presented with a previous history of laminitis; to have elevated ACTH concentrations; or to be receiving pergolide treatment (Table 2). Apart from noting that all three Hanoverian horses were in the slow group, and that these were among the heaviest horses in the study (with a mean BWT of 614 kg), the breed range was too diverse to be able to identify any clear association between breed and rate of improvement.

Several variables were investigated based on visual and radiographic examination of the foot, to determine if the rate of improvement in clinical signs was associated with the frequency or size of visible radiographic abnormalities observed on d 0; or with signs of chronic laminitis, including divergent laminar rings, convex or flat sole, widened white line, and/or change in hoof wall angle (Table 3).

**Table 3:** Visual and radiographic examination of the hoof in horses at first presentation with hyperinsulinaemia-associated laminitis, partitioned according to their rate of improvement, as judged by a clinical scoring method

Variable	Fast improvement (***n*** = 27)	Slow improvement (***n*** = 10)	***P*** value^**a**^
Signs of chronic laminitis on the forefeet^b^, n (%)	16 (59.3)	6 (60)	1.000
Rotation of the distal phalanx, n (%)	24 (88.9)	9 (90)	1.000
Distal displacement of the distal phalanx (sinker), n (%)	11 (40.7)	5 (50)	0.716
Decreased sole depth at the tip of the distal phalanx, n (%)	7 (25.9)	3 (30)	1.000
Lamellar wedge formation, n (%)	6 (22.2)	3 (30)	0.679
Osteo-remodelling, n (%)	5 (18.5)	3 (30)	0.655
Radiolucent/gas lines, n (%)	5 (18.5)	2 (20)	1.000

^a^Odds-ratio test

^b^Signs include divergent laminar rings, convex or flat sole, widened white line, and/or change in hoof wall angle

Evidence of chronic laminitis was reported in a high proportion (59%) of cases overall. In qualitative terms, rotation of the distal phalanx was judged to occur most frequently (reported in 90% of cases), while osteo-remodelling and radiolucent/gas lines were reported least often (in 22 and 19% of cases, respectively). All events were reported with similar frequency between the slow and fast-recovery groups, with no significant differences observed.

In quantitative terms, there was a strong correlation between the angle of rotation observed for the left and right foot (R^2^ = 0.719, *P* < 0.001), consistent with usual presentation of laminitis as bilateral lameness. There was also a clear association between the angle of rotation and the incidence of previous laminitis episodes, such that the median (IQR) rotation angle in horses with chronic laminitis (10.2°, 5.6–15°) was more than twice that seen in horses that showed no evidence of previous laminitis (4°, 3–8.6°; *P* = 0.006).

However, there was no such relationship with the rate of improvement in clinical signs, as angle of rotation in the slow improvement group (6.4°, 3.88–11.52°) was not larger than in the fast recovery group (7.1°, 2.85–12.95°; *P* = 0.792). Even when horses with signs of previous laminitis were excluded from the analysis, the median angle of rotation in the slow improvement group (3.6°, 2.73–7.75°) was still not larger than that in the fast improvement group (4.9°, 3–8.6°; *P* = 0.744). Thus, the slow rate of improvement in clinical signs was not associated with, or predicable from, a larger degree of rotation on first presentation.

**Fig. 3:**
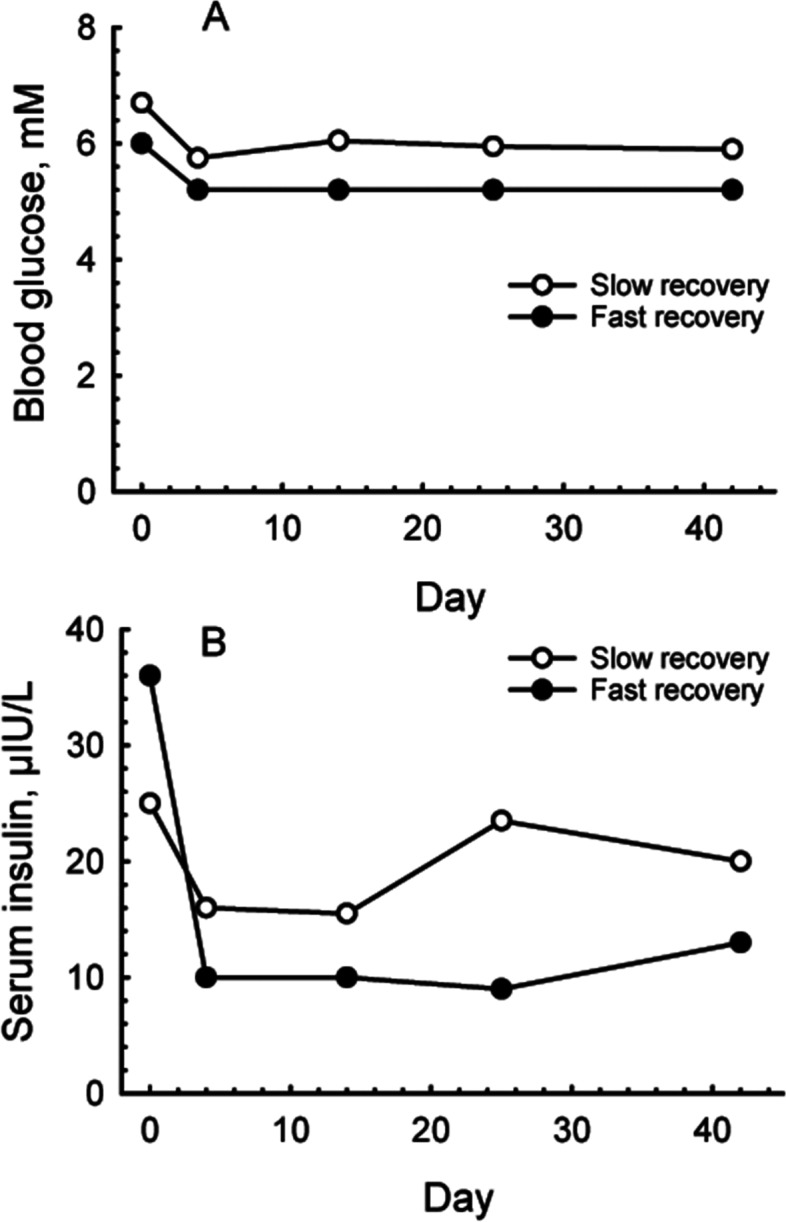
Median blood glucose (**A**) and serum insulin (**B**) concentrations, measured over 42 days in 27 horses and ponies that recovered quickly and 10 that recovered slowly from hyperinsulinaemia-associated laminitis

Finally, the data were analysed to compare the concentrations of glucose and hormones between the two groups (Table 4, Fig. 3). Although glucose concentrations remained within the normal range in both groups, they were 11 to 16% higher in the slow improvement group on d 4 (*P* = 0.019), d 14 (*P* = 0.032) and d 25 (*P* = 0.014). Insulin concentrations were elevated to a greater extent, being 51 to 55% higher in the slow improvement group on d 14 (*P* = 0.022) and d 25 (P = 0.034) and remained close to significance on d 42 (*P* = 0.062). Log insulin concentrations on d 0 were also associated with the frequency of previous laminitis episodes, being higher (*P* = 0.042) in horses with evidence of chronic laminitis (1.73 ± 0.12 μIU/mL), than in horses with no evidence of chronic laminitis (1.4 ± 0.10 μIU/mL). There was no difference between the groups in the concentrations of leptin or ACTH.

**Table 4:** Median (interquartile range) blood glucose and hormone and concentrations measured over 42 days in 37 horses during a rapid (n = 27) or slow (n = 10) improvement following hyperinsulinaemia-associated laminitis. All samples were collected following an overnight fast, except on d 0

Analyte	Improvement rate	Days after diagnosis				
		0	4	14	25	42
Glucose, mM	Fast	6 (5.20–7.0)	5.2 (4.9–5.6)*	5.2 (4.6–5.7)*	5.2 (4.9–5.6)*	5.2 (4.7–5.7)
	Slow	6.7 (5.26–7.73)	5.75 (5.33–6.75)	6.05 (4.88–6.68)	5.95 (5.28–6.98)	5.9 (5–6.75)
Insulin, μIU/mL	Fast	36 (12–130)	10 (5–19.0)	10 (5–16)*	9 (5–20)*	13 (5–19)†
	Slow	25 (13.25–110)	16 (8.75–86.3)	15.5 (11–45.3)	23.5 (9.75–50.8)	20 (12–38)
ACTH, pg/mL	Fast	31 (17.4–52.6)	24.1 (12.8–38.8)	22.6 (14.1–48.2)	28.5 (14.9–64.7)	37.9 (17.4–117)
	Slow	18.0 (14.2–39.6)	17.5 (14.1–37.1)	15.7 (13.4–29.3)	17.9 (15.8–35.0)	22.6 (16.9–58.7)
Leptin, ng/mL	Fast	21.0 (16.5–25.8)	15.9 (11.9–28.6)	15.6 (12.4–28.1)	18.8 (9.3–27.7)	17.4 (12–26.7)
	Slow	18.4 (15.6–42.8)	20.1 (14.2–34.2)	19 (14.6–37.4)	17.3 (14.4–43.2)	16.6 (13.6–47.2)

† *P* < 0.1, *P < 0.05, comparisons are between fast and slow groups on the same day, Mann-Whitney Rank Sum Test

## Discussion

This study has demonstrated that while the clinical signs of laminitis can improve markedly and within a short time (14 days) when horses with HAL are managed appropriately, some horses (about 1 in 4 in the present study) recover much more slowly and may show signs of laminitis that wax and wane over several weeks, despite the best efforts of the attending veterinarian. Understanding the factors associated with a slow recovery could improve the treatment options for these individuals. As far as we are aware, ours is one of the few studies to evaluate the variables associated with clinical improvement, and thus complements previous studies which have focussed on the risk factors for HAL [2, 7, 15].

Of all the variables measured, blood glucose concentrations showed the highest probability of an association with a slow improvement. However, because the difference between the median glucose concentrations was relatively small, it is unlikely this analyte will be useful as a prognostic marker. Similarly, high insulin concentrations were also associated with a slow improvement, and with evidence of previous laminitis, but although a difference in median values of 51 to 55% may be clinically significant, the use of this hormone as a prognostic marker is also problematic, due to the large variation in the values observed. Nevertheless, our findings are consistent with previous studies indicating that elevations in blood glucose and insulin are involved in the pathogenesis of HAL [6, 19–21]. Furthermore, an association has been demonstrated previously, between insulin concentrations and the severity of laminitis upon presentation, as well as between the change in insulin concentrations and change in laminitis (Obel) grade, during recovery [22].

We cannot rule out the possibility that the degree of foot pain might have influenced glucose and insulin concentrations, which were measured 24 h after pain medication had been withheld. However, based on ACTH concentrations, there was no evidence that the hypothalamic-pituitary-adrenal (HPA) axis was activated to a greater extent in the slow recovery group, which might have been expected if this group has suffered more pain than the fast-recovery group. Notwithstanding the possible influence of pain, it is clear that an association between glucose, insulin and rate of improvement exists, regardless of whether the former variables are causative or symptomatic.

The reason for a small but a persistent elevation in blood glucose in the animals that were slow to improve, is not known. Until d 14, all but one of the horses in this group were fed hay only. However, we cannot rule out possible differences in hay quality, and in particular it would be useful to know the non-structural carbohydrate (NSC) content of the diet for each horse, as HAL can be induced in ID ponies by feeding a diet that has a high NSC content [19]. Another possibility is that these animals suffered more extreme ID, and that some might have developed insulin resistance, but a dynamic intravenous test would have been required to establish this. Of note, is that the median insulin concentration in this group was not higher than in the fast improvement group on d 0. In terms of a practical application for this finding, our data suggest that if the observed metabolic differences do contribute to a slow recovery, then glucose and/or insulin-lowering agents such as velagliflozin [21] could be useful in these cases.

The lack of any direct association between ACTH and improvement rate is not surprising. While high ACTH concentrations are associated with PPID [23], and PPID is associated with a higher risk of laminitis [23], this is very likely because animals with PPID can exhibit high insulin concentrations [15, 20]. Furthermore, horses and ponies with elevated ACTH (indicative of PPID), and those already diagnosed with PPID and being treated with pergolide, appeared in the fast and slow improvement groups with similar frequency.

Similarly, the lack of association between leptin concentrations and improvement rate was not unexpected. Although leptin concentrations are positively correlated with body condition in horses [24] and may also be associated with ID (through obesity-related insulin resistance [25]), there is no evidence that leptin plays a direct role in the pathogenesis of laminitis. The lack of data for another important adipokine, adiponectin, is a limitation of this study, as adiponectin has been correlated directly with laminitis risk [15], and this may be worth further investigation.

Among the morphological variables, the only variable that was statistically significant was age, with a difference of 4.5 years between the median values for each group. A younger median age in the slow improvement group is difficult to explain in physiological terms, and this variable also needs further investigation.

Body weight was thought to be a relevant morphologic parameter, as it has been associated with the extent of pathological changes in sepsis related laminitis [26] and suggested to account for the faster rate of laminitis onset in horses compared with ponies, when they are infused with insulin [9]. Similarly, it was once thought that generalized obesity was an important risk factor for the development of laminitis [11, 27]. Animals with a wide range of body weight were enrolled in the present study, and although the horses and ponies that recovered slowly were 8% heavier on average, this difference was not statistically significant. Even though obesity may have several detrimental effects on the horse, the present results support the theory that generalised obesity is not in itself a major factor in determining the rate of recovery from the disease, based on the distribution of horses graded as ‘very fat’.

We observed that the proportion of males in the fast improvement group was more than twice that in the slow group, but the total number of animals was small and this seemingly large quantitative difference was not statistically significant. Therefore, while we cannot conclude that sex influences the probability of a fast recovery, this should be investigated further.

Finally, although radiographic changes in the hoof were observed in 90% of the horses, the speed of improvement could not be predicted based on the type or frequency of these observations. This conclusion held for both qualitative and quantitative assessments. Radiographs can provide valuable information about disease severity under certain circumstances, but our previous experience of using radiographic data in cases of HAL has revealed three important limitations. First, radiographs may reveal changes that were present before the current bout of laminitis, and this is important because there is a high frequency of recurrence in HAL [4]. In fact, this was likely to have been the case in a large proportion of horses used in the current study, and in 35% of ponies used in an earlier study when HAL was induced experimentally [19]. Secondly, the radiographic changes may be minimal (e.g. a movement in the distal phalanx of only 1–2 mm) despite clear clinical signs of pain and lameness. Thirdly, horses may show a full recovery in terms of clinical signs, with no resolution of the radiographic changes after many weeks. Notwithstanding these limitations, we acknowledge that a more detailed investigation of radiographic changes such measuring the extensor process to coronary band distance, dorsal hoof wall to distal phalanx distance, or the “lucent zone” width in the dorsal hoof wall, could be helpful in future studies.

There were two main limitations of this study. First, the number of horses and ponies in the slow improvement group was relatively small, and a larger population might have revealed additional factors that influence recovery. Secondly, although the veterinarians operated under a strict and detailed protocol and recorded exactly how each animal was treated and fed, several variables such as hay quality and feed intake were not measured accurately. However, instead of being a tightly controlled animal house experiment, the study was intentionally conducted under field conditions, and the variability that occurred may have been important in revealing a range of factors that determine the improvement rate from HAL in a real-world setting.

## Conclusions

In summary, this study has successfully identified three variables (age, blood glucose and insulin) that are associated with the rate of improvement from HAL in a clinical field setting. Further research is warranted to investigate factors that influence recovery in more depth, to demonstrate the potential value of glucose/insulin lowering agents in subjects that are slow to recover, and to seek other variables that may be useful diagnostic and prognostic indicators.

## Methods

### Animal selection and characteristics

This observational study initially employed 41 control animals that has been selected at random to participate in a larger field trial involving 80 horses. Experienced equine veterinarians were recruited by the study sponsor from 16 clinics across Germany and trained in all methods applicable to the study, including the ‘modified Obel’ laminitis scoring system developed by Meier et al. [18]. This training included the provision of detailed documentation and assessment of video recordings. The performance variables associated with this scoring system have been reported previously [18]. One veterinarian from each clinic was designated as the primary investigator and a second veterinarian was designated as the co-investigator. Whenever possible the same investigator performed the examinations and was responsible for reviewing the data collected.

Over a 12-month period these veterinarians identified 80 naturally-occurring cases of suspected HAL in a wide range of horse and pony breeds. Animals identified for the study were those that had suffered a bout of laminitis within the past 48 h, achieving a clinical laminitis grade of 1 or more using the traditional Obel system, and a score of ≥5 on a 12-point scale using the modified Obel system [18].

Horses with identifiable triggers indicative of non-HAL, such as severe colic, colitis, metritis, contralateral weight-bearing etc., were not considered for the study. Observations that led to a suspicion of HAL included a body condition score of ≥4 on a scale of 0 to 5 developed by Carroll and Huntington [28]; fat in specific sites such as the nuchal crest, rump, supraorbital region, prepuce or mammary region; a previous diagnosis of ID based on elevated resting glucose or insulin concentrations, or dynamic testing; or a diagnosis of PPID made on the basis of ACTH values and clinical signs.

Among these 80 cases, 39 were selected at random to participate in a follow-up clinical study and the remaining 41 (which served as a control group) were assigned for a detailed investigation, including hormone analysis. Dynamic testing for ID was not performed due to a perceived risk of conducting this type of test during a laminitis episode.

Of the 41 horses considered for the present study, 4 cases were not confirmed as HAL, based on the observation that their basal insulin concentrations measured on d 0, 4, 14, 25 and 42, never exceeded 8.5 μIU/ml. This lower threshold was taken from a previous study in which 75% of horses above the threshold developed laminitis when subsequently fed a high NSC diet [19]. Of the remaining 37 horses, 31 cases were designated as confirmed HAL on the basis that insulin concentrations exceeded 20 μIU/ml. This is the diagnostic threshold for suspected ID if consistent with clinical signs, according to the most recent recommendations of the Equine Endocrinology Group [10]. Six horses that fell between these thresholds were also included in this study, as they all had multiple clinical signs consistent with ID and HAL, including cresty necks, fat deposition at specific sites and evidence of previous laminitis episodes. Table 5 presents the demographic and morphological characteristics of the final study cohort.

**Table 5:** Characteristics of horses and ponies presenting with hyperinsulinaemia-associated laminitis

Variable	Category
Total subjects, n	37
Horses, n	19
Ponies, n	18
Geldings, n	15
Mares, n	22
Age, median (IQR), yr	17 (12.5–20)
Estimated body weight, median (IQR), kg	394 (237–480)
Body condition (score), n	
Good (3)	7
Fat (4)	21
Very fat (5)	9
History of laminitis, n	21
Treatment with pergolide, n	9
Elevated ACTH^a^, n	14

^a^According to seasonally adjusted norms

Although 14 animals in the cohort were subsequently found to have elevated ACTH concentrations (according to seasonally-adjusted norms [29]), only 9 had been diagnosed previously with PPID (on the basis of elevated ACTH and clinical signs) and were being treated with pergolide. As all the treated animals had been on a constant dose of pergolide for at least 6 weeks prior to the study, with no change in medication during the study, they were included in the cohort. Nevertheless, the potential confounding effects of elevated ACTH and/or pergolide treatment were examined during the data analysis.

### Study procedures

The cases were managed at their owner’s properties by the equine veterinarians who followed a detailed study protocol. On the day of presentation (d 0), all horses underwent a clinical examination, and their medical and dietary history were recorded. Body condition was scored [27], and body weight was estimated using a weight tape according to the method described by Wagner et al. [30], where estimated body weight (kg) = heart girth^2^ (cm^2^) x body length (cm) / 11,880 (cm^3^). The feet were checked for signs of chronic laminitis (divergent laminar rings, convex or flat sole, widened white line, or change in the hoof wall angle), and lateral radiographs were taken of both forefeet. The angle between the hoof wall and the distal phalanx was measured by two blinded observers for each foot using these radiographic images. A laminitis examination was also performed [18].

From d 0 to at least d 9, all the horses were subjected to rest/confinement in a stable, their diet was restricted to hay only, and they were fitted with supportive pads and bandages (which were removed for subsequent laminitis assessments). Dietary, exercise and other interventions beyond this date, and other concomitant treatment such corrective hoof care, were prescribed at the discretion of the attending veterinarian and were recorded to ensure there were no marked disparities in the level or nature of care provided. With the exception of pergolide, no medications that can alter endocrine function, such as metformin, levothyroxine, and corticosteroids were used during or before the study. The short-acting analgesics flunixin or phenylbutazone were allowed at the discretion of the attending veterinarian but were withheld 24 h before each laminitis assessment. Long-acting analgesics or other medications (such as tranquilizers) that could mask the signs of lameness during laminitis examinations, were prohibited. Other concomitant treatments that were prohibited throughout the study include heparin and acepromazine.

Further clinical and laminitis evaluations took place on d 4, 9, 14, 25 and 42. Blood samples were also collected by jugular venepuncture on each of these days (except d 9). Prior to each visit the horses were not allowed to feed ad libitum overnight, but were permitted a small amount of hay only. The blood was collected into plain tubes for serum, or EDTA and NaF tubes for plasma. The samples were kept chilled until they were processed, normally within 8 h. The samples were analysed by one commercial veterinary diagnostics laboratory (Vet Med Laboratories GmbH, Ludwigsburg, Germany) for glucose (hexokinase assay), insulin and ACTH (Advia Centaur immunoassay system), and by a second diagnostics laboratory (Clinic for Cattle, University of Veterinary Medicine Hannover, Foundation, Germany) for leptin (Millipore XL-85 K RIA). All assays were validated for use in horses.

### Data analysis

The data were subjected to a Shapiro-Wilk test for normality. Few of the variables conformed to a normal distribution, even after several types of transformation (log, reciprocal, square root), and so multivariable analysis was not possible. Instead, non-parametric analyses were performed. The Friedman test for repeated measures ANOVA on ranks was used to identify changes in ACTH, glucose, insulin, and leptin concentrations over time, and when a significant difference was identified, the values at each timepoint were compared to those on day 0 using Dunnett’s test. If a significant difference was identified other than on d 0, then multiple pairwise comparisons were made using Tukey’s test.

A visual inspection of the pattern of improvement in clinical signs in individual horses indicated that while most of the animals had shown a marked improvement by d 14, there were some clear exceptions. Therefore, the horses were separated *post-hoc* into two groups, a fast-improvement and a slow-improvement group, as detailed in the results section. An Odds ratio test was used to compare the frequency of subjects in each group, according to age, body weight, sex, body condition score, history of laminitis, elevated ACTH, or pergolide treatment. The hormone and glucose concentrations in these groups were compared using a Mann-Whitney Rank Sum Test. The results are reported as medians (interquartile range; IQR). All statistical analyses were performed using Sigmaplot™ Version 13.0^1^ Significance was set at *P* < 0.05.

### Fate of the animals

The horses remained with their owners at the end of the study.

## Data Availability

The full data sets used and analysed during this study are not publicly available due to the fact that individual client owned horses may be identified, but are available from the corresponding author in a redacted form, or with permission of the owners, on reasonable request.

## Abbreviations

ACTH: Adrenocorticotrophic hormone
ANOVA: Analysis of variance
d: Day
EDTA: Ethylenediaminetetraacetic acid
GLP-1: Glucagon-like peptide-1
h: Hour(s)
HAL: Hyperinsulinaemia-associated laminitis
ID: Insulin dysregulation
IQR: Interquartile range
NaF: Sodium fluoride
NSC: Non-structural carbohydrate
PPID: Pituitary *pars intermedia* dysfunction
QUT: Queensland University of Technology
RIA: Radioimmunoassay
SE: Standard error
VICH: Veterinary International Conference on Harmonization (of Technical Requirements for Registration of Veterinary Medicinal Products)

## Footnotes

1 Sysstat Software Inc., San Jose, California, USA
